# Insights into the Functioning of the D-amino Acid Transaminase from Haliscomenobacter Hydrossis via a Structural and Spectral Analysis of its Complex with 3-Aminooxypropionic Acid

**DOI:** 10.32607/actanaturae.27496

**Published:** 2024

**Authors:** A. K. Bakunova, I. O. Matyuta, A. Yu. Nikolaeva, K. M. Boyko, A. R. Khomutov, E. Yu. Bezsudnova, V. O. Popov

**Affiliations:** Bach Institute of Biochemistry, Research Center of Biotechnology of the Russian Academy of Sciences, Moscow, 119071 Russian Federation; National Research Center “Kurchatov Institute”, Moscow, 123182 Russian Federation; Engelhardt Institute of Molecular Biology, Russian Academy of Sciences, Moscow, 119991 Russian Federation; Department of Biology, Lomonosov Moscow State University, Moscow, 119234 Russian Federation

**Keywords:** transaminase, enzymatic catalysis, crystal structure, inhibitor, 3-aminooxypropionic acid

## Abstract

Pyridoxal 5’-phosphate-dependent enzymes play a crucial role in nitrogen metabolism. Carbonyl compounds, such as O-substituted hydroxylamines, stand out among numerous specific inhibitors of these enzymes, including those of practical importance, because they react with pyridoxal 5’-phosphate in the active site of the enzymes to form stable oximes. O-substituted hydroxylamines mimic the side group of amino acid substrates, thus providing highly potent and specific inhibition of the corresponding enzymes. The interaction between D-amino acid transaminase from bacterium *Haliscomenobacter hydrossis *and 3-aminooxypropionic acid was studied in the present work. The structural and spectral analyses of the complex of this transaminase with 3-aminooxypropionic acid allowed us to clarify some features of the organization and functioning of its active site and illustrate one of the mechanisms of inhibition by the specific substrate, D-glutamic acid.

## INTRODUCTION

Pyridoxal 5’-phosphate (PLP)-dependent transaminases (aminotransferases, TAs, EC [2.6.1.X]) catalyze the transfer of an amino group from amino acid or amine to keto acid or ketone to form a new amino acid/amine and keto acid/ketone [1, 2]. Enzymatic transamination is a sequential double displacement process involving the intermediate transfer of an amino group to the PLP cofactor, giving rise to pyridoxamine 5’-phosphate, which acts as an amino group donor in the second half-reaction. Two substrates (the amino acid and keto acid) sequentially bind to the same active site region; all the reaction stages are reversible [1, 3]. Transaminases have successfully been used as stereoselective catalysts of amino group transfer for asymmetric amination of compounds carrying a keto group and for separation of chiral primary amines [4, 5]. Only two of the seven types of polypeptide chain folding of PLPdependent enzymes (fold types I and IV) are characteristic of transaminases. The mechanism of catalysis and the structure of a functional dimer transaminase have been intensively studied for fold type I (*S*)-selective transaminases. Fold type IV transaminases have been characterized to a lesser extent. Interestingly, both (*S*)-selective (transaminases of branched L-amino acids) and (*R*)-selective enzymes (D-amino acid transaminases and (*R*)-amine:pyruvate transaminases) have been found among them. It is (*R*)-selectivity that has re-kindled interest in the study of fold type IV transaminases over the past decade.

**Fig. 1:**
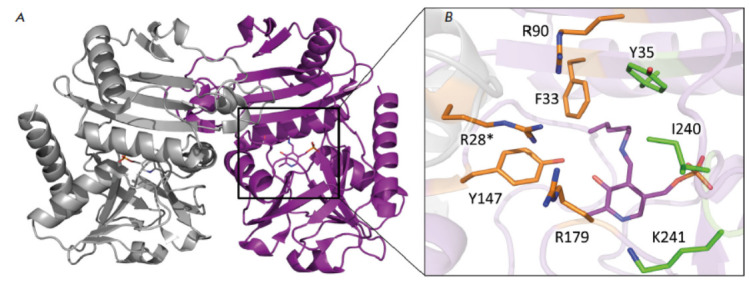
The overall structure of TA_Halhy. (*A*) TA_Halhy homodimer; (*B*) active site of TA_Halhy. O-pocket residues are colored in orange; P-pocket residues are colored in green. The PLP molecule is colored in purple. * – residues of the adjacent subunit of the function dimer

Carbonyl compounds, including hydroxylamine derivatives, are typical inhibitors of PLP-dependent enzymes. One of the algorithms for designing high-efficient and selective inhibitors from hydroxylamine esters (R-ONH_2_) is to use derivatives mimicking the side group structure of amino acid substrates. The functional groups in the O-substituted hydroxylamine radical ensure substrate-like binding of the inhibitor to the enzyme active site, while the reactive aminooxy group interacts with PLP to form oxime. This approach allows one to produce inhibitors with a nanomolar binding constant not only for TAs such as aspartate aminotransferase [6, 7], but also for decarboxylases specific for glutamic acid [8], ornithine [9], and arginine [10]. It is noteworthy that the use of hydroxylamine-containing analogs of putrescine and agmatine enables selective inhibition of closely related ornithine and arginine decarboxylases [10]. The structural similarity of external aldimine, one of the intermediates in PLP-catalyzed amino acid transformations, and PLP oxime formed by substrate-/product-like hydroxylamines was confirmed for the first time by X-ray diffraction analysis of the enzyme inhibitor complexes of aspartate aminotransferase [6, 7]. Later, similar studies were performed for gamma-aminobutyric acid transaminase [11], ornithine decarboxylase [12], and D-amino acid transaminase [13]. The structures of the complexes of PLP-dependent enzymes with such hydroxylamine derivatives make it possible to analyze the structure, as well as the features, of substrate binding and functioning of the enzyme active site. In the present study, this approach was employed for investigating D-amino acid transaminase from *Haliscomenobacter hydrossis *(TA_Halhy). TA_Halhy belongs to the group of fold type IV transaminases and efficiently catalyzes transamination reactions between D-amino acids and α-keto acids; specific activity in the reaction between D-glutamate and pyruvate in 50 mM potassium phosphate buffer (pH 8.0) hits the record high values for TAs: 380 ± 10 μmol/min per mg of protein at 40°C [14, 15]. The structure of this enzyme, dimer being its functional unit, has been identified (*Fig. 1A*). Like for the studied fold type IV TAs, the active site of TA_Halhy can be subdivided into two parts (the O- and P-pockets); the amino acid residues of these pockets are involved in substrate binding, thus being responsible for the stereospecificity of catalytic transformation (*Fig. 1B*). TA_Halhy stands out among the known fold type IV TAs by featuring four positively charged functional groups in its active site (side groups of amino acids Arg28*, Arg90, Arg179, and Lys241) [14, 16] (*Fig. 1B*).

**Fig. 2:**
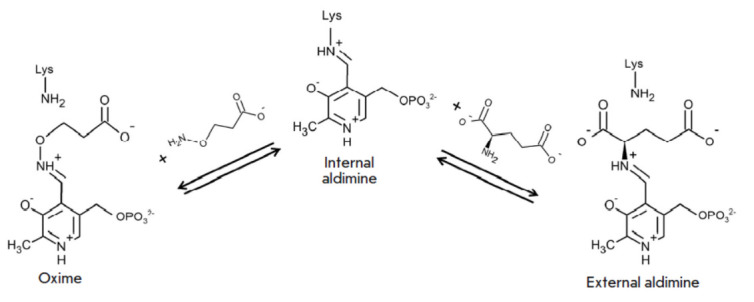
Scheme of the interactions of internal aldimine (holoenzyme) of TA_Halhy with 3-aminooxypropionic acid (an oxime is formed) and D-glutamic acid (an external aldimine is formed)

The results of an analysis of the structure of the TA_Halhy complex with an inhibitor, D-cycloserine, suggested that the side groups of Arg28* and Arg179 residues are involved in substrate binding [17]. Since we failed to identify the structure of the TA_Halhy complex with substrates (because of the high efficiency of amino acid conversion in TA_Halhy-catalyzed reactions, crystallization with substrates yields an apoenzyme), research into the structure of the TA_Halhy active site was continued by analyzing the interaction between the enzyme and 3-aminooxypropinic acid (an analog of the D-glutamic acid substrate) by UV/Vis spectrophotometry and X-ray diffraction analysis. 3-Aminooxypropionic acid was shown to interact with PLP in the active site of TA_Halhy to form an oxime that mimics the external aldimine of PLP and D-glutamic acid (*Fig. 2*); therefore, successful crystallization and solving the complex’s structure make it possible to identify the functional groups involved in the binding of this specific substrate.

## MATERIALS AND METHODS

**Expression and purification of recombinant TA_Halhy**

Purified active recombinant TA_Halhy was prepared according to the procedure described previously [14]. The purity and homogeneity were controlled electrophoretically in denaturing polyacrylamide gel (SDS-PAAG). TA_Halhy concentration was determined spectrophotometrically at 280 nm.

**Spectral analysis**

The PLP form of TA_Halhy (holoenzyme) was prepared by incubating the enzyme (2.5 mg/mL, or 74 μM) in 50 mM potassium phosphate buffer (pH 8.0) with excess PLP (700 μM) in the presence of 10 mM α-ketoglutarate during 30 min. Low-molecular-weight components were removed from the holoenzyme by transfer into 50 mM potassium buffer (pH 8.0) using a HiTrap Desalting column (Cytiva, USA) equilibrated in the same buffer.

3-Aminooxypropionic acid (10 mM) was added to the holoenzyme (0.85 mg/mL, or 25 μM) in 50 mM potassium phosphate buffer (pH 8.0), and the mixture was allowed to stand for 60 min. The protein fraction was separated from the low-molecular-weight components on the HiTrap Desalting column. The fraction of low-molecular-weight components was also obtained by ultrafiltration using a centrifugal concentrator (30 kDa MWCO, Millipore, USA). The absorption spectra were recorded in 50 mM potassium phosphate buffer, pH 8.0, using an Evolution 300 UV-Vis spectrophotometer (Thermo Scientific, USA).

**Substrate inhibition**

The TA_Halhy-catalyzed transamination reaction was conducted in 50 mM potassium phosphate buffer, pH 8.0, at 40°C with substrates D-alanine (40 mM) and α-ketoglutarate or D-glutamate and pyruvate (2.5 mM) supplemented with 30 μM PLP, 0.33 mM NADH, and 5 μg/mL lactate dehydrogenase (specific activity, 200 μmol/min per mg of protein). Lactate dehydrogenase was stable under the conditions of the transamination reaction. No heat inactivation of TA_Halhy was observed at 40°C [14].

**Preparing crystals of the TA_Halhy complex with oxime of PLP and 3-(aminooxy)propanoic acid**

Crystals of the complex were prepared by co-crystallization of the TA_Halhy holoenzyme with 12 mM 3-aminooxypropinic acid in the presence of excess PLP (6 mM) under the following conditions: 0.1 M bis-Tris-propane, pH 5.5, 0.2 M MgCl_2_, 25% PEG 3350.

**Collection and analysis of the diffraction data. Structure solution and refinement**

Right before the X-ray diffraction experiment, TA_Halhy crystals were placed into a cryosolution containing 25% (v/v) glycerol, along with counter- solution ions; the crystal in a loop sample holder was then frozen in liquid nitrogen vapor. The XRD data recorded at 100 K on the Protein Factory of the synchrotron radiation source at Research Center “Kurchatov Institute” were analyzed using the Dials software [18] from the CCP4 software package [19]. *Table 1* shows the statistics of the recorded dataset. The structure was solved by the molecular replacement method using the MOLREP software [20]. The REFMAC5 software was used for refinement [21]. The structure of the holo form of D-acid transaminase from *H. hydrossis *(PDB ID 7P7X) was used as a starting model. Visual analysis of structural data was performed using the Coot [22] and PyMOL Molecular Graphics System, Version 4.6, software (Schrödinger, USA).

**Table 1:** Statistics for data collection, analysis, and crystallographic refinement of the structure of the TA_Halhy complex with the oxime formed by PLP and 3-aminooxypropionic acid

Study object	TA_Halhy complex
X-ray source	National Research Center “Kurchatov Institute”
Wavelength, Å	0.74503
Temperature, K	100
Analysis	
Space group	C2
Unit cell parameters	a = 88.77 Å, b = 71.23 Å, c = 52.55 Å; α = γ = 90°, β = 101.26°
Resolution, Å	35.34–1.70 (1.73–1.70)
Number of independent reflections	32789 (1795)
Completeness, %	94.9 (98.6)
Rmeas, %	10.1 (54.6)
Mean I/σ(I)	11.4 (1.9)
CC1/2, %	99.1 (60.2)
Refinement	
Rwork, %	16.6
Rfree, %	21.0
Overall average B-factor	17.9
Average B-factor for protein	16.8
Average B-factor for ligands	16.5
Average B-factor for solvent	26.2
Number of non-hydrogen atoms	
Total	2607
Protein	2275
Ligands	23
Solvent	309
Root mean square deviations	
Bond lengths, Å	0.01
Bond angles, °	1.67
Ramachandran plot	
Most favored, %	98.2
Allowed, %	1.8
PDB ID	8YRV

## RESULTS AND DISCUSSION

**Spectral analysis of interactions between TA_Halhy and 3-(aminooxy)propanoic acid**

**Fig. 3:**
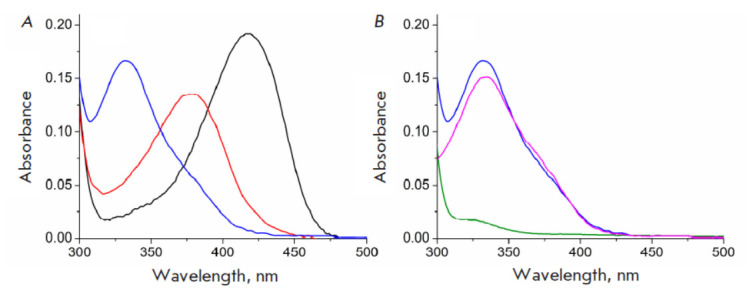
Spectral changes in TA_Halhy (25 μM) upon addition of 3-aminooxypropionic acid in 50 mM potassium phosphate buffer, pH 8.0, at 25°C: (*A*) the absorption spectra of holoenzyme TA_Halhy before (black), immediately after addition (red), and after 1 h of incubation with 3-aminooxypropionic acid acid (blue); (*B*) the absorption spectra of holoenzyme TA_Halhy after 1 h of incubation with 3-aminooxypropionic acid acid (blue) followed by exchange in 50 mM potassium phosphate buffer, pH 8.0 (green); the absorption spectrum of the low-molecular-weight fraction collected by ultrafiltration (pink)

*Figure 3* shows the spectra of the holoenzyme TA_Halhy (25 μM) in 50 mM potassium phosphate buffer, pH 8.0, immediately after the addition of 10 mM 3-aminooxypropionic acid and incubation at 25°C for 1 h. The observed changes attest to the formation of the oxime of PLP and 3-aminooxypropinic acid within the active site of TA_Halhy (the spectrum with λmax = 380 nm), and release of the oxime from the active site of the solution (the spectrum with λmax = 333 nm corresponds to the spectrum of the oxime of PLP and H2NOR in the solution [23]). *Figure 3B* demonstrates that after the transfer to a new buffer and one hour of incubation, the spectrum of TA_Halhy corresponded to the apoenzyme (without PLP and its adducts). Holoenzyme was formed, and TA_Halhy activity was fully restored, after PLP was added to the resulting apoenzyme solution.

The rapid formation of the oxime of PLP and 3-aminooxypropionic acid in the active site of TA_Halhy (*Fig. 3A*, spectrum with λmax = 380 nm) is consistent with knowledge that Schiff bases (in this case, internal aldimine) react with O-substituted hydroxylamines much faster compared to the respective aldehyde [24]. The efficiency of TA inhibition by O-substituted hydroxylamines depends on the structural similarity of the radical of O-substituted hydroxylamine and the side group of the amino acid substrate, as well as the strength of PLP binding to the enzyme’s active site [8, 9, 10, 25, 26]. Thus, aspartate aminotransferase forms strong oximes with aminooxyacetic and 3-aminooxypropionic acids, which mimic external aldimines with substrates, as well as aspartic and glutamic acids. The carboxylic groups of the inhibitors act as anchors and ensure additional binding of oximes to the enzyme’s active site. When excess hydroxylamines are removed, oximes of PLP do not get released from the enzyme active site and adding excess PLP does not restore enzyme activity, either [6]. Contrariwise, TA_Halhy has low affinity to PLP (*K*d = 1.9 ± 0.3 μM [16]) and the oxime of PLP is easily released from the active site (*Fig. 3*). Similar dissociation was observed upon interaction between TA_Halhy, D-cycloserine [17], and phenylhydrazine [16], thus attesting to the open active site of TA_Halhy, which seems to retain its open conformation during catalytic transformations [14, 16]. Dissociation of the complex with the oxime leads to the accumulation of the apoenzyme (*Fig. 3B*). Adding PLP to the TA_Halhy complex with oxime causes enzyme reactivation; an active holoenzyme is formed as a result of complex dissociation and release of the oxime from the active site yielding the apoenzyme, followed by interaction between the apoenzyme and the added PLP: therefore, inhibition by 3-aminooxypropionic acid is reversible. The reactivation of TA_Halhy after PLP had been added was consistent with the apoenzyme stability that had been demonstrated previously [15].

**Fig. 4:**
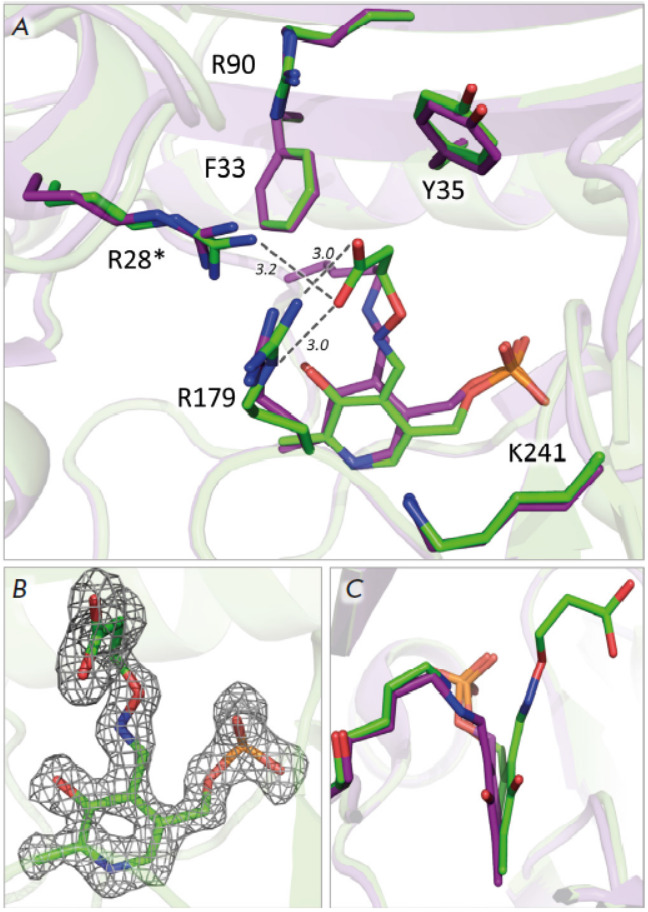
The active site of the complex of TA_Halhy with 3-aminooxypropionic acid: (*A*) superposition of the structures of the complex (green; PDB ID 8YRV) and holoenzyme TA_Halhy (purple; PDB ID 7P7X), distances are given in angstroms and depicted with dashed lines; (*B*) the “omit” electron density map (Fo-Fc) of the oxime of PLP and 3-aminooxypropionic acid is depicted at the 3σ level; (*C*) superposition of the PLP in the holoenzyme (purple) and in the complex with oxime (green)

We successfully crystallized the TA_Halhy complex with the oxime of PLP and 3-aminooxypropionic acid in the active site. A set of diffraction data has been collected in the XRD experiment; the structure of the TA_Halhy complex has been solved and refined. The structures of the holoenzyme and complex with the oxime are well-superposed (RMSD for Cα atoms is 0.31). Differences are mostly observed for the positions of loops. Importantly, the carboxyl group of the oxime of PLP and 3-aminooxypropinic acid is located in the O-pocket, although in fold type IV D-amino acid transaminases the side group of the substrate binds within the P-pocket, while the O-pocket binds the α-carboxyl group of substrates (D-amino acid or keto acid), which forms hydrogen bonds with the functional groups of the active site [27, 28]. In the resulting structure, the carboxyl group of the oxime forms hydrogen bonds with the guanidine groups of Arg28* and Arg179 residues. The Arg90 and Lys241 residues are not involved in the binding of the carboxyl group; the side group positions in all the aforementioned residues remain unchanged. The geometry of the holoenzyme active site is retained in the structure of the complex with oxime (*Fig. 4A*).

**Fig. 5:**
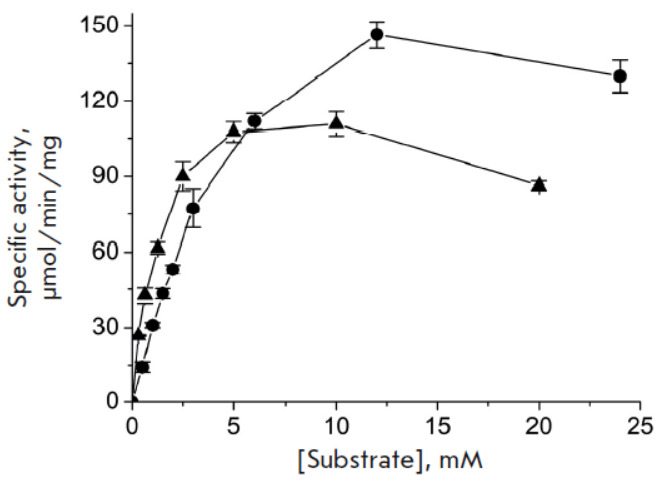
Substrate inhibition of TA_Halhy in the transamination reaction between D-glutamic acid and 2.5 mM pyruvate (●) and between α-ketoglutarate and 40 mM D-alanine (▲) in 50 mM potassium phosphate buffer, pH 8.0, at 40°C. Bars denote the standard deviation

The observed adduct position in the active site of TA_Halhy mimics substrate inhibition rather than the formation of an external aldimine with D-glutamic acid as a specific substrate. Substrate inhibition is known to accompany transaminase catalysis because of the similarities in substrate (amino acids and keto acids) binding. TA_Halhy is inhibited by D-glutamic acid and α-ketoglutarate at substrate concentrations as low as millimolars (*Fig. 5*). At least two inhibition mechanisms are known: (1) D-glutamic acid binds to the active site containing a pyridoxamine 5’-phosphate instead of the keto substrate and (2) the position of the α-carboxyl group is occupied by the γ-carboxyl group of D-glutamic acid or α-ketoglutarate. This very type of binding is observed in the complex (*Fig. 4A*). This nonproductive inhibitory binding is consistent with the high observed dissociation constant of the TA_Halhy complex with D-glutamic acid determined using the half-reaction method (*K*d = 1.8 ± 0.4 mM [29]).

It is also worth mentioning that the position of the PLP molecule in the complex structure s changes: in the oxime complex, the PLP molecule is tilted towards the active site entrance by 18° along the N1–C6 axis (*Fig. 4B,C*). The change in the cofactor position is observed as the internal aldimine is converted to an external one (*Fig. 2*) [13, 27]. These findings support the hypothesis that the cofactor, in the form of an internal aldimine, is under the stress relieved when an external aldimine (rupturing of a covalent bond with the side group of the catalytic lysine residue [30]), or oxime in the case of 3-aminooxypropionic acid, is formed. Interestingly, the active site of TA_Halhy remains open after oxime formation, which is proved by the fact that the oxime is released into the solution after one hour of incubation of the enzyme in the presence of excess 3-aminooxypropionic acid (see above). Open configuration of the active site was observed previously for TA_Halhy in complexes with phenylhydrazine and D-cycloserine; Open configuration of the active site was also observed for the homologous D-amino acid transaminase from *Aminobacterium colombience *in complexes with D-glutamic acid and 3-aminooxypropionic acid [13], as well as for the canonical D-amino acid transaminase from *Bacillus *sp*. YM-1 *in complex with D-alanine [27]. In other words, stereoselective transamination in D-amino acid transaminases seems to take place without active site closure (separation from the solvent), unlike in the case of fold type I transaminases [7].

## CONCLUSIONS

The following conclusions can be drawn from the study of the interaction between D-amino acid transaminase holoenzyme from *H. hydrossis *and 3-aminooxypropionic acid: (1) inhibition by 3-aminooxypropinic acid is reversible; (2) the active site of transaminase remains open after substrates/inhibitors binding; (3) coordination of the carboxyl group of the oxime in the O-pocket confirms that the Arg28* and Arg179 residues are involved in substrate binding; however, the observed position of the oxime corresponds to substrate inhibition, when a substrate (α-ketoglutarate and D-glutamic acid) binds nonproductively (via the γ-carboxyl group in the O-pocket of the active site), and the reactive amino group of the substrate faces away from PLP and the side group of the catalytic lysine residue.

## Abbreviations

NADH: reduced nicotinamide adenine dinucleotide
PLP: pyridoxal 5’-phosphate
TA: transaminase
TA_Halhy: D-amino acid transaminase from Haliscomenobacter hydrossis
